# Orthodontic Implementation of Machine Learning Algorithms for Predicting Some Linear Dental Arch Measurements and Preventing Anterior Segment Malocclusion: A Prospective Study

**DOI:** 10.3390/medicina59111973

**Published:** 2023-11-09

**Authors:** Aras Maruf Rauf, Trefa Mohammed Ali Mahmood, Miran Hikmat Mohammed, Zana Qadir Omer, Fadil Abdullah Kareem

**Affiliations:** 1 Department of Pedodontics, Orthodontics and Preventive Dentistry, College of Dentistry, University of Sulaimani, Sulaimaniyah 46001, Iraq; aras.rauf@univsul.edu.iq (A.M.R.); trefa.ali@univsul.edu.iq (T.M.A.M.); 2 Department of Basic Sciences, College of Dentistry, University of Sulaimani, Sulaimaniyah 46001, Iraq; miran.mohammed@univsul.edu.iq; 3 Department of Pedodontics, Orthodontics and Preventive Dentistry, College of Dentistry, Hawler Medical University, Erbil 44001, Iraq; zana.qadir@hmu.edu.krd

**Keywords:** machine learning, linear dental arch, preventing malocclusion

## Abstract

*Background and Objectives*: Orthodontics is a field that has seen significant advancements in recent years, with technology playing a crucial role in improving diagnosis and treatment planning. The study aimed to implement artificial intelligence to predict the arch width as a preventive measure to avoid future crowding in growing patients or even in adult patients seeking orthodontic treatment as a tool for orthodontic diagnosis. *Materials and Methods*: Four hundred and fifty intraoral scan (IOS) images were selected from orthodontic patients seeking treatment in private orthodontic centers. Real inter-canine, inter-premolar, and inter-molar widths were measured digitally. Two of the main machine learning models were used: the Python programming language and machine learning algorithms, implementing the data on k-nearest neighbor and linear regression. *Results*: After the dataset had been implemented on the two ML algorithms, linear regression and k-nearest neighbor, the evaluation metric shows that KNN gives better prediction accuracy than LR does. The resulting accuracy was around 99%. *Conclusions*: it is possible to leverage machine learning to enhance orthodontic diagnosis and treatment planning by predicting linear dental arch measurements and preventing anterior segment malocclusion.

## 1. Introduction

Artificial intelligence (AI) has recently been incorporated into many aspects of life, including medical ones. The imitation of human activities by computers to accomplish a task in an easier and faster way is called artificial intelligence, which is a very general term that describes imitation. Machine learning (ML) is the term that is usually associated with AI. Machine learning is the process of enabling software applications to predict outcomes more accurately by using a variety of sample data. Specialized algorithms generate mathematical models that generalize certain patterns to predict decisions without having been explicitly programmed for that individual task [1].

Nowadays, with increasing computing power, such algorithms have been enabled to be used for complex tasks, and a revolution has been initiated by this technology in some areas of medicine [2].

Promising potential for supporting medical decisions was observed with some applications of AI, while in some cases, it even outperformed experienced clinicians in terms of diagnostic efficiency [3,4,5,6].

Researchers and clinicians have experienced some remarkable approaches for orthodontic diagnostics, therapy planning, and the prognosis of treatment outcomes. Artificial intelligence is involved in the segmentation of anatomical or pathological structures in diagnostic images, the identification of reference points, and the support of decision making in a variety of orthodontic cases. The benefits of routinely using AI are related to time savings for certain diagnostic procedures and enhanced quality management. Nevertheless, like with any kind of technological aid, it is necessary for AI users to become aware of its possible limitations and potential implications [7].

Artificial neural networks (ANN) were first used only in orthodontics to decide whether or not to extract a tooth, anticipate a change in the curvature of the lip, and forecast the shape of the arch [8,9,10]. It was discovered that ANN-based model analyses were more precise than traditional methods were [11].

According to Ali et al., 2021, artificial intelligence systems with neural network machine learning would be useful as an accurate method in orthodontics for determining the presence of unerupted teeth, and its performance was achieved through processes such as proper input data selection, preferable generalization, and appropriate organization [12]. ANNs were created as a new and accurate approach for determining the presence of unerupted teeth using the MATLAP program (R2020a v 9.8/2020). This network achieved a high degree of correlation between the sum of the mesiodistal breadth of premolars, canines of the target, and the actual output.

Accuracy, safety, and simplicity are some key requirements for a predictive approach before it can be used in the thorough case analysis used in modern orthodontic practice [12]. The segmentation and classification of teeth is a fundamental step in orthodontic treatment planning. AI has also been applied in a variety of methods, including radiography and full-arch 3D digital optical scans [13,14]. Cui et al. suggested numerous artificial intelligence (AI) methods for autonomously segmenting teeth using a digital teeth model scanned using a 3D intraoral scanner [13] and CBCT images [14,15]. They segmented alveolar bone as well as teeth, and their efficiency exceeded that of radiologists (i.e., it was 500 times quicker). The method also performs effectively in difficult instances with varied dental defects, according to the research [14]. Given the current technological advances, it is not unrealistic to anticipate that artificial intelligence (AI) will become an important element of orthodontic diagnoses and treatment planning in the near future. Although AI will most likely not be able to replace the expertise and experience of human professionals in the near future, it will most likely be able to help practitioners, functioning as a quality-assuring component in orthodontic patient care [16].

No previous study was conducted using the main ML (machine learning) models, which are k-nearest neighbor and linear regression, using Python programming language, for predicting the arch width from the upper incisor width as a preventive measure. 

## 2. Materials and Methods

### 2.1. Registration for Study

The University of Sulaimani’s College of Dentistry’s ethics committee granted this study’s clearance (199 on 10th of January 2023).

### 2.2. Sample

Four hundred fifty intraoral scan (IOS) images from class I malocclusion patients seeking orthodontic treatment in private orthodontic clinics were chosen in which there was not more than 3 mm of crowding or spacing, all permanent teeth were present except 3rd molars, no previous orthodontic treatment or orthognaphic surgery were performed and a mean age of 20 ± 7 years. All the IOS pictures were obtained by an experienced orthodontist who had completed hundreds of practice scans using an intraoral scanner (Medit i 700 Wireless) in accordance with the manufacturer’s instructions. Digital measurements of the actual inter-canine, inter-premolar, and inter-molar widths were taken from the scanned pictures using Medit design software version 2.1.2.

### 2.3. Study Protocol

The aim of the study is to use artificial intelligence to predict the arch width as a preventive measure to avoid future crowding in growing patients or even for adult patients seeking orthodontic treatment as a tool for orthodontic diagnosis and treatment plans.

In this work, some of the main ML (machine learning) models are used using the Python programming language. In addition to machine learning algorithms, this work implements the data in k-nearest neighbor and linear regression.

Before starting the direct implementation of ML algorithms, it is important to go through some important steps, which are called feature engineering. This starts with cleaning the collected data from imbalanced quantities and magnitudes among the features. The large difference in quantities for the input data affects the performance of value prediction and introduces significant errors when the output is tested with different evaluation metrics. Those metrics are the MAE (mean absolute error), MSE (mean square error), and R2 score (coefficient of determination). These metrics are ML evaluations of model performance in terms of the accuracy and error they produce. Furthermore, it is important to give suggestions to fine-tune and update the model parameters to make better output predictions in the next steps.

Another critical step in feature engineering is checking the input features for a missing row or single value under a column. There are many techniques for filing missing values, and this work used the method of obtaining the mean value of columns and put the result inside the empty cell where it is found. Furthermore, the impact of having empty cells in any machine learning model results in low accuracy because important values are missing in the ML equations. This is because any ML algorithm consists of a set of mathematical equations, and missing data cause the parameters of the calculation to be nulls or zeros. In our dataset, none of the missing values are found, which means all the columns are filled with appropriate values regarding the column feature.

The last step of feature engineering is detecting and removing the outliers in the data, especially among the input feature columns. Outliers make the result of a prediction have very high differences in range, leading to increased prediction errors, and the accuracy usually decreases below 70%. However, in our dataset, very few outliers are detected, and they don’t make a vast impact on the ML model that is used.

Then, the next important step starts, which is the implementation of the machine learning models on the dataset. In this work, the models that are used concentrate on supervised learning, as the collected data are labeled with known input features (upper right central width, upper left central width, upper left lateral width, and upper right lateral width) and target output columns (U/I-M, L/I-M, U/I-pre, L/I-pre, and U/ant).

The ML models are KNN (k-nearest neighbor) and LR (linear regression). The main reason for using different models is to show the different ranges of the accuracy of predictions and choose the best model as the standard and generalize it for more data, especially for those implemented in actual practice.

For the machine learning implementations, some important steps are followed using Python programming language. The first step is reading the CSV file containing the dataset, by using the pandas python library. After loading the data, it is required to split them into two parts: the training and testing sets. By using the train_test_split method from the Sklearn library, the train set is split. The split works by dividing the dataset and giving the train set 80% of the dataset and the test set 20% of the dataset. This split means that the model should first go into the training process, learn from the data distribution based on model equations, and then give the resulting output. Once the ML is trained on the data, after that test, the test sets are entered into the model to check the accuracy and error of the model. The test set works on evaluating model performance; in case the output accuracy of the test set of the model is low, hyperparameter tuning starts and tunes the parameters. Then, the model trains again on new parameters that are updated and tests the result again until it reaches the relevant output accuracy that is acceptable with a low error.

**Figure 1:**
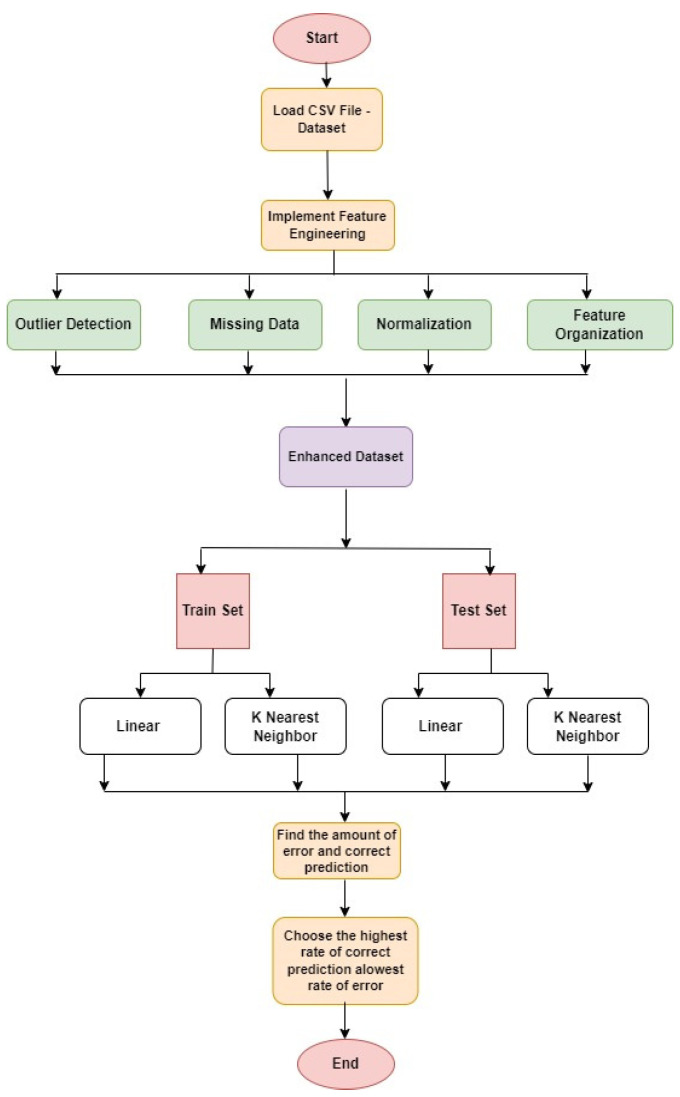
Flowchart of the proposed algorithm used in the research.

Furthermore, this is an important step to follow before finalizing the model for real-time implementations. Figure 1 shows the flowchart of the process used in this paper. The following is the pseudo-code algorithm for the proposed work.

### 2.4. Proposed Algorithm Steps

**Algorithm 1:** Algorithm proposed machine learning technique **Step 1**: Read CSV file using pandas library;**Step 2**: Perform feature engineering;   Step 2.1: Check missing values;   Step 2.2: Check for outliers;   Step 2.3: Check the correlation among features;**Step 3**: Split the dataset into two parts, training and testing, with 20% for testing from the total dataset. **Step 4**: Implement the training data in linear regression and K-Nearest Neighbor and calculate the accuracy using the MSE and R2_score.**Y^^^** = $\sqrt{\sum_{i=1}^{n}{\left(xi-yi\right)}^{2}}$ **KNN—Equation formula**
**Y^^^: Predicted value**

***xi***
**: Actual point value 1**

***yi***
**: Actual point value 2**

**Y^^^ = B_0_ + B_1_X_i_**

**Linear Regression—Equation**

**Y^^^: Predicted value**

**B_0_**
**: Y intercept**

**B_1_**
**: Slop Coefficient**

**X_i_**
**: Independent actual value**

**MSE =**

1n∑i=1n(yi−y^i)2


**Mean Square Error—Equation**

***n***
**: number of total data points**

***yi***
**: Actual value**

***y^i***
**: Predicted value**
**Step 5**: Choose the height accuracy with less error.
**End**


Furthermore, the main aim of this research is to include all the required columns in the ML models and predict the four main output values, which are here to put the exact name of the columns as the output.

## 3. Results

**Figure 2:**
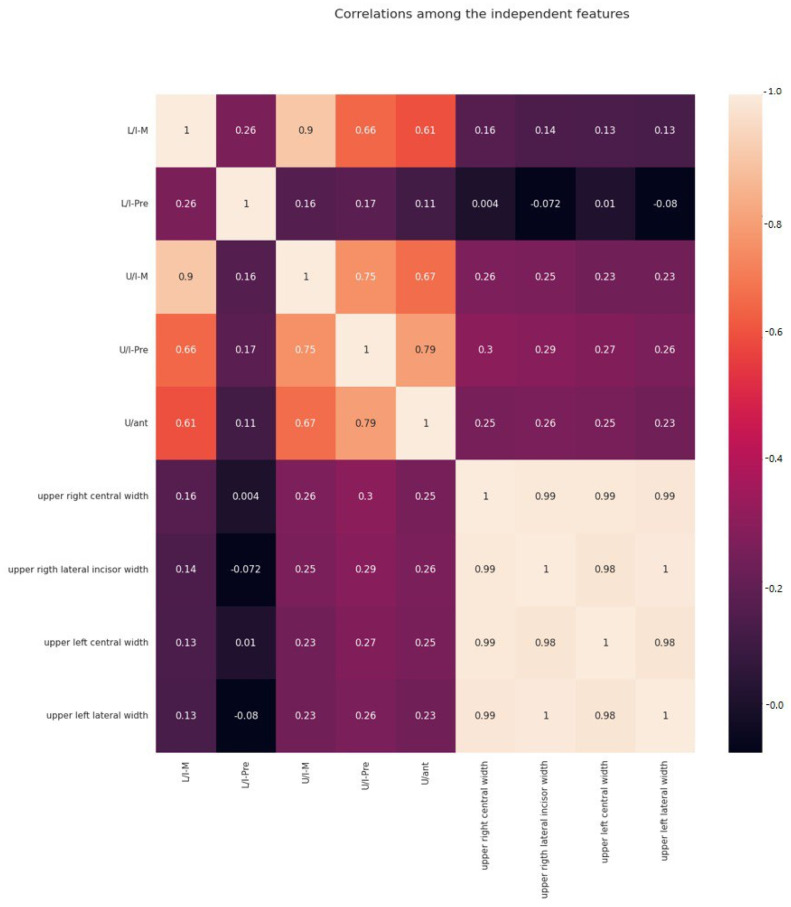
A chart showing the correlations among the independent features.

**Figure 3:**
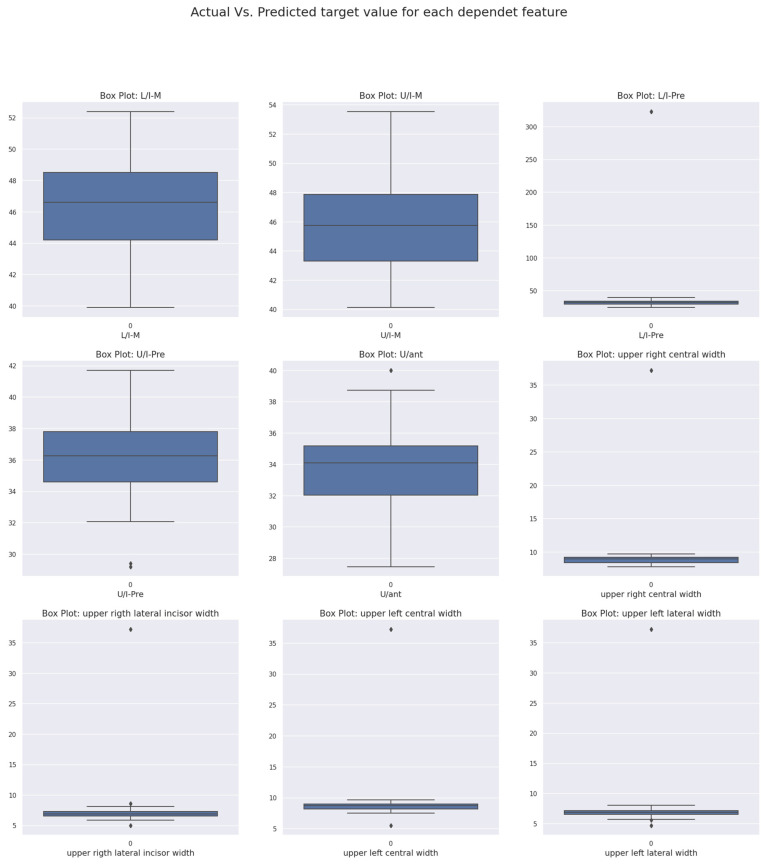
Box charts for independent features that shows there are no effective outliers.

The main descriptions of the dataset show that all the columns presented in the dataset play an important role in the predictions made with ML algorithms. They have relevant correlations among the columns, which are input features for ML algorithms. For example, the feature upper right central width has a 0.99 correlation with the columns upper left central width, upper right, and left lateral width, whereas the correlation of upper right lateral width with upper left lateral width is 1, and with upper left central width, it is 0.98. Additionally, the correlation of upper left central width with upper right and left lateral width is 0.98. Finally, upper left lateral width has a 0.98 correlation with upper left central width. As they are chosen as input features for the ML algorithms, these high correlations are important because they make better predictions with a lower accuracy rate as shown in Figure 2 representing the correlation among columns. Additionally, the dataset columns have been tested for enormous outliers. Fortunately, the result shows that very few outliers are detected, and they do not have to decrease the accuracy of predictions. Furthermore, they are not very far from the range of quartiles. The input features (upper right central width, upper left central width, upper left lateral width, and upper right lateral width) have very few outliers, with no more than two data points in each of them, so they do not cause any drawbacks in the data point distribution. Moreover, in case of the output columns, which are U/I-M, L/I-M, U/I-pre, L/I-pre, and U/ant, although in columns U/I-M and L/I-M no outliers are detected, in the columns there are only two data points considered outliers in U/I-pre and one data point found in L/I-pre and U/ant. Furthermore, they do not have any efficiency in the ML model, because the total amount contained in the dataset that is used is around 400, which is too few to have a high impact on the results (as shown in Figure 3).

After the dataset is implemented in the two ML algorithms, linear regression and k-nearest neighbor, the evaluation metric shows that KNN gives better prediction accuracy than LR does. The resulting accuracy is around 99%. The output was the prediction of five values, which are L/I-M, U/I-M, L/I-Pre, U/I-Pre, and U/ant. This implementation works on two main values: the actual value from the dataset that is used and the prediction value that the algorithm produces according to the ML algorithm equations (Algorithm 1).

**Figure 4:**
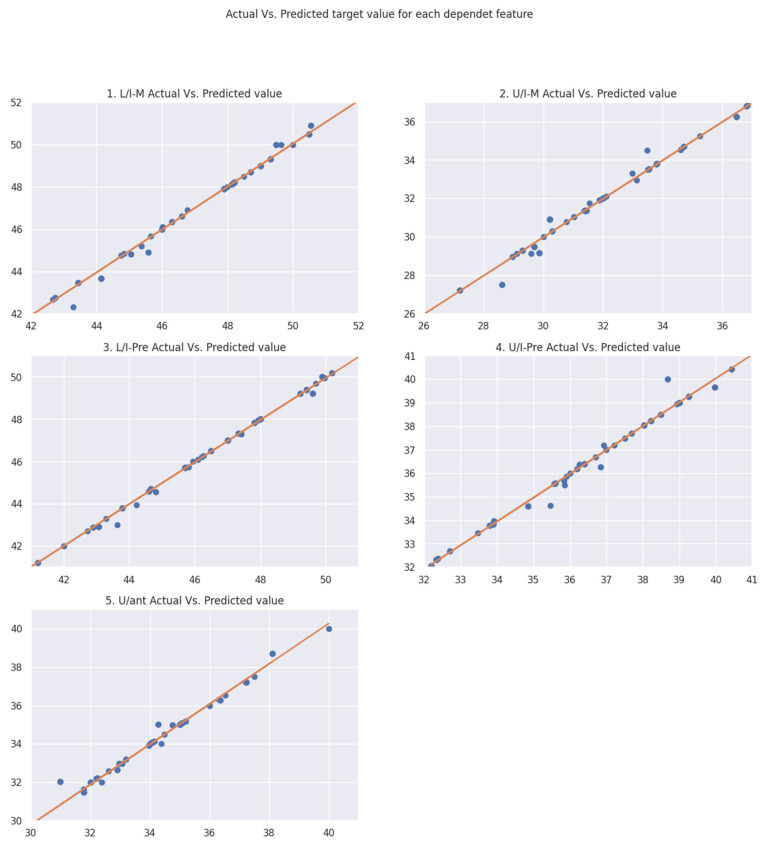
The linear plot of actual vs. predicted target values.

Figure 4 shows the linear distribution points of both actual and predicted values for each target feature separately. It is clear that most of the points fit on the slope line, which indicates that the amount of correction in the correct prediction is high and reliable; furthermore, the sloping line demonstrates the two points, which were the predicted value and the actual value. As is clear, most of the points fit on the line, which means the actual and predicted values are very close to each other, and this means that the ML algorithm has the ability to give predictions of values that are correct and tend less to be faulty results. In addition, the data that are plotted on the graph are the test data, which are split from the original dataset into train and test sets (80% for the train set and 20% for the test set).

Hence, from the prediction result of column L/I-pre, it can be seen that most of the points lie on the sloping line; only a few points remain out of the line, but they are very close to the line. Therefore, they do not create a high difference between the actual and the predicted value.

The same procedure can be noticed in the other columns. However, for most of the points, the differences between them and the sloping line are not more than one to two points. For example, the column U/I-Pre has a point located at y coordinate −40, while the line coordinate in that location is 39.

Furthermore, the location of the points and the slope of the line have a high impact on the algorithm’s accuracy. This means that, with very far data points from the line, the results have low accuracy. This is because each ML algorithm has an equation called the cost function that calculates the errors, and the error here means the distance between the actual point and the predicted point. With that, with fewer data points that are far from the line, better accuracy is achieved and less errors are produced. That is, the algorithm will become powerful and have the ability to predict any future value.

## 4. Discussion

Orthodontics is a field that has seen significant advancements in recent years, with technology playing a crucial role in improving diagnosis and treatment planning. Implementing machine learning algorithms in orthodontics, specifically for predicting linear dental arch measurements and preventing anterior segment malocclusion, is a promising area of research.

This prospective study aimed to leverage machine learning to enhance orthodontic diagnosis and treatment planning by predicting linear dental arch measurements and preventing anterior segment malocclusion. It is important to follow a rigorous research methodology, ensure data accuracy, and prioritize ethical considerations to produce meaningful results that can benefit orthodontic practice. Because Pont’s index is not trustworthy in forecasting arch widths for the Kurdish population, alternative formulae to estimate inter-molar, inter-premolar, and anterior arch widths were recommended [17].

Dental arch measurements can involve complex relationships and patterns. Machine learning algorithms can capture these intricate patterns, which might be challenging to model using traditional statistical methods.

Machine learning models can learn from vast datasets, which can lead to highly accurate predictions. In orthodontics, precision is crucial, as even small errors in measurements can affect treatment outcomes.

Once trained, machine learning models can automate the process of measuring and predicting dental arch parameters. This can save significant time for orthodontists, improve efficiency in treatment planning, and help identify early signs of malocclusion or other dental issues, allowing for timely intervention and potentially preventing more severe problems down the line. Machine learning can tailor treatment plans to individual patients based on their unique dental arch measurements and characteristics. This personalization can lead to more effective and patient-centric care. Moreover, it can uncover subtle correlations and factors contributing to dental arch measurements that might not be apparent through traditional analysis. This can lead to new insights in orthodontic research.

Machine learning models can be updated and improved as more data become available. This adaptability ensures that predictions remain relevant and accurate over time.

While there may be an initial investment in developing and implementing machine learning models, in the long run, they can lead to cost savings by streamlining diagnosis and treatment planning processes.

The use of artificial intelligence (AI) in medicine and dentistry is growing in importance. It has potential in many settings where advanced technology may aid in and improve the lives of people [18].

Some of the most common algorithms used in orthodontics include artificial neural networks (ANN), convolutional neural networks (CNN), support vector machines (SVM), and regression methods [19].

To determine if extractions were necessary for a patient’s treatment plan, Peilini et al. employed an ANN. The anchoring patterns were also taken into account. The artificial neural network accurately predicted successful extractions (94%) and optimal anchoring usage (92%) from treatment plans. These findings suggest that orthodontists can use ANN to create more targeted treatment programs [11]. Detecting TMJ osteoarthritis may be easiest with the use of deep learning neural networks, according to the study. Osteoarthritis (OA) of the TMJ is a progressive disability that affects between 5% and 12% of the population and worsens with time. Discomfort in the jaw joint (TMJ) ranks as the second most common musculoskeletal issue. The primary objective is to identify TMJ dysfunction prior to the onset of morphological degeneration. The study by Bianchi et al. [20,21,22] used CBCT scans of the TMJ in addition to blood and saliva testing to reach conclusions. Cephalometric points were placed on cephalometric radiographs using an ANN in the work by Muraev et al. Comparisons were made between the ANN’s placement accuracy and that of three distinct groups of doctors: the most seasoned, the most regularly practicing, and the least seasoned. The results demonstrated that ANNs were as excellent as a trained dentist are at constructing cephalometric points and, in certain instances, might even be more accurate than freshly qualified medical experts [23]. The application of ANNs might also aid in pinpointing certain stages of maturation. In the study by Kök et al., cephalometric and hand–wrist radiographs were taken from patients aged 8 to 17. Cervical vertebrae were used to determine the phases of development and gender, with an accuracy value of 94.27% [24].

Digital diagnostic methods have advanced significantly during the last ten years. Clinicians that use these applications may find it easier to diagnose, plan treatments, and make decisions. These programs are incredibly helpful since they are dependable, quick, and capable of automatically carrying out the work with an efficiency comparable to that of seasoned professionals. For orthodontists with little expertise, these models may be a great resource. The small datasets utilized for training and testing these models and the dependability of the data due to the fact that they were gathered from a single hospital, institution, or piece of equipment are some problems with the majority of these models. Therefore, further creativity in this area is required for improved generalizability and dependability [25].

## 5. Conclusions

Based on accurate data manipulation and sophisticated ML training, artificial intelligence facilitates orthodontic diagnosis and treatment planning by predicting linear dental arch measurements, and eventually helps in preventing anterior segment malocclusion. Moreover, K Nearest Neighbor was presented to have more more prediction accuracy than linear regression was in the Python programming language of ML models. By harnessing the power of machine learning algorithms, researchers and clinicians can now offer more precise and personalized treatment options for patients.

## Data Availability

Data are available from the corresponding author upon request.
